# The Fourth Trimester: Embracing the Chaos of the Postpartum Period

**DOI:** 10.31486/toj.24.5043

**Published:** 2024

**Authors:** Tabitha M. Quebedeaux, Stacey Holman

**Affiliations:** ^1^ Department of Obstetrics and Gynecology, Division of Maternal Fetal Medicine, Louisiana State University Health Sciences Center, School of Medicine, New Orleans, LA; ^2^ General Obstetrics and Gynecology, Louisiana State University Health Sciences Center, School of Medicine, New Orleans, LA

*Mrs Z is a 34-year-old, African American, morbidly obese patient who has just given birth to her fourth child at 37 weeks gestational age. Her delivery timing was not planned as she was sent to Labor and Delivery for elevated blood pressures and subsequently underwent emergent cesarean delivery because of nonreassuring fetal status. On postpartum day 1, her blood pressures remain elevated, and she is diagnosed with gestational hypertension. Mrs Z is overwhelmed by this new hypertension diagnosis and must prepare for her recovery from unexpected abdominal surgery. She is also concerned about how she will care for her young children while her spouse works two jobs to support their family.*

*On postpartum day 2, she states that she must be discharged because her spouse is assigned to work, and her children will be alone at home. The medical team reviews her clinical recovery, notes only mildly elevated blood pressures, and ensures she is without symptoms of worsening hypertensive disease. Mrs Z is given instructions about warning signs, particularly for severe preeclampsia, and a follow-up appointment is scheduled for a blood pressure check in 3 days. She misses that postpartum visit and does not answer her phone when the doctor attempts to call. She calls the team on postpartum day 6 with complaints of increasing swelling in her lower extremities and a pounding headache. She has borrowed her neighbor's blood pressure cuff and reports a reading of 150/105. She is instructed to come directly back to the hospital. She states she will locate a sitter for her children and will ask a neighbor to bring her to the hospital.*

*Six hours later, Mrs Z is brought to the hospital by emergency medical services after her oldest child found her seizing in their home. She is intubated, and computed tomography imaging shows subarachnoid hemorrhage. During the next 24 hours, the patient experiences persistent seizure activity despite medical management and ultimately suffers cardiac arrest. Care is withdrawn on postpartum day 7.*

Pregnancy-related deaths are defined as maternal deaths during pregnancy, delivery, and within the first year postpartum. Mrs Z's clinical presentation and sudden death vividly portray data reported by maternal mortality review committees across the United States from 2017-2019. In 36 US states, pregnancy-related death disproportionally impacted women of color and was found to be preventable in more than 80% of cases.^1^ Maternal death is most likely to occur during the postpartum window (53%), and cardiac conditions are the most common cause of maternal death in non-Hispanic Black patients.^1^

The American College of Obstetricians and Gynecologists (ACOG) and the Society for Maternal-Fetal Medicine (SMFM) have made valiant efforts to address the issues of maternal cardiovascular disease in pregnancy and its impact on postpartum maternal health. ACOG and SMFM have focused efforts on increasing obstetrics provider education with an extensive review of maternal cardiovascular pathophysiology,^2^ updated guidelines highlighting the urgency of hypertensive treatment in pregnancy,^3,4^ a checklist for the discharge of a hypertensive postpartum patient,^5^ and a reimagining of the continuum of care from pregnancy to lifelong medical care.^6^

Despite these efforts, the National Vital Statistics System showed a continued increase in maternal mortality rates from 2019 to 2021.^7^ The maternal mortality rate increased from 20.1 deaths per 100,000 live births in 2019 to 32.9 deaths per 100,000 live births in 2021, and non-Hispanic Black mothers were 2.6 times more likely to die than non-Hispanic White mothers.^7^ These findings are mirrored in the *Louisiana Pregnancy-Associated Mortality Review* investigation of 15 pregnancy-related deaths in 2020.^8^ The leading causes of pregnancy-related death were cardiomyopathy and cardiovascular conditions, and 53% of the deaths occurred within 42 days of delivery. Thirteen of the 15 women who died a pregnancy-related death in Louisiana during 2020 were Black.^8^

ACOG has acknowledged the risk of maternal death during the postpartum period by reintroducing the *fourth trimester* into the paradigm of pregnancy care.^6^ Traditional postpartum care often ended at the routine 6-week follow-up for all deliveries. Only patients with surgical interventions, hypertension diagnoses, or mood disturbances were sometimes scheduled for a more frequent 1-week follow-up appointment. The current recommendation is that all postpartum women have contact with a provider within 3 weeks of delivery, with a continuation of care provided through 12 weeks postpartum.^6^ Studies have shown that while obstetrics providers recognize the importance of this new paradigm, there are significant barriers to achieving these goals.^9,10^ Obstetrics providers and their patients often agree on the challenges faced in postpartum care, including overbooked clinic schedules, insurance limitations, childcare, transportation, lack of provider continuity, and knowledge gaps.^9,10^

Mrs Z represents the nearly 40% of postpartum patients who do not present for follow-up care.^6^ This high percentage cannot be attributed to medical noncompliance alone. Instead, patients face continued challenges during this phase of care, including nonmedical social determinants of health. These barriers often hinder the ability of patients to address acute health concerns. The changing paradigm of postpartum care involves bringing health care to the patient within her community through innovative strategies.

Because maternal mortality is deeply impacted by hypertension/cardiovascular disease, SMFM recommends remote blood pressure monitoring for postpartum patients who have hypertension during pregnancy.^5^ In the hospitals that we serve, patients have access to a program called Babyscripts, a comprehensive remote monitoring program developed for pregnancy and the postpartum period. The program aims to provide access to health care by addressing barriers and improving health equity outcomes. Patients like Mrs Z are provided with education at discharge and a Bluetooth-enabled blood pressure monitor to alert medical providers to escalating blood pressures. Babyscripts connects the patient to a nurse and alerts the patient when to seek medical care. A study that investigated the impact of postpartum home blood pressure monitoring reported that 20% of patients recorded one or more significant elevations in blood pressure.^11^ Alerted medical providers were then able to adjust medications and identify which patients needed to return for medical care.^11^ We have noted success with this program in our own community by identifying patients prior to their first follow-up visit who needed to return to the hospital for urgent care.

Another opportunity to empower patients during the chaotic postpartum phase of care is the availability of a program called Family Connects.^12^ This model of care employs a universal approach to reach families with a free home visit by a nurse who conducts maternal and newborn screenings. It is available to birthing families in Orleans Parish and is supported by the New Orleans Health Department. Patients are typically visited about 3 weeks after delivery to identify areas of need and provide a human connection to community resources. The nurse also assists with infant bonding and screens for any signs of health concerns for both mom and baby. The visiting nurse can arrange critical medical care with obstetrics providers, pediatricians, and mental health support.

In our community, social determinants of health are the foundation of care in this visit. Barriers that have been addressed include transportation, access to healthy food and diapers, creating safe spaces in the home, and communication with clinics for setting up appointments. Here are typical comments from our patients about this experience:

*“It is convenient and provides great support.”*

*“I was happy about it since my recovery didn’t go as planned and having a nurse was helpful because I had so many things going on with myself and my baby.”*

Mrs Z's story is a tragic reminder of the patient stories that continue to be told too frequently in the United States. The majority of maternal deaths are patients who live in urban areas near medical centers, yet their access to care remains limited. An individualized postpartum care plan is one of the many necessary steps to prevent poor obstetrical outcomes. A humanistic approach to care encourages us to move beyond focusing on systemic issues in medicine. Obstetrics providers must embrace the chaos and work with patients to optimize care during this time.

We must not look at Mrs Z as simply a postpartum hypertensive patient but instead as a mother of four who is doing her absolute best to care for her family and then herself. She is the center of her own community. Ultimately, by using humanistic approaches and acknowledging our patients’ struggles, we can replace chaos with education, empowerment, and healing.
